# Krüppel-Like Factors 9 and 13 Block Axon Growth by Transcriptional Repression of Key Components of the cAMP Signaling Pathway

**DOI:** 10.3389/fnmol.2020.602638

**Published:** 2020-11-12

**Authors:** José Ávila-Mendoza, Arasakumar Subramani, Robert J. Denver

**Affiliations:** Department of Molecular, Cellular and Developmental Biology, The University of Michigan, Ann Arbor, MI, United States

**Keywords:** Krüppel like factor, hippocampus, cyclic AMP, neurite outgowth, axon regeneration

## Abstract

Krüppel-like factors (KLFs) are zinc finger transcription factors implicated in diverse biological processes, including differentiation of neural cells. The ability of mammalian neurons to elongate axons decreases during postnatal development in parallel with a decrease in cAMP, and increase in expression of several *Klf* genes. The paralogous KLFs 9 and 13 inhibit neurite outgrowth, and we hypothesized that their actions are mediated through repression of cAMP signaling. To test this we used the adult mouse hippocampus-derived cell line HT22 engineered to control expression of *Klf9* or *Klf13* with doxycycline, or made deficient for these Klfs by CRISPR/Cas9 genome editing. We also used primary hippocampal cells isolated from wild type, *Klf9*^–/–^ and *Klf13*^–/–^ mice. Forced expression of *Klf9* or *Klf13* in HT22 changed the mRNA levels of several genes involved with cAMP signaling; the predominant action was gene repression, and KLF13 influenced ∼4 times more genes than KLF9. KLF9 and KLF13 repressed promoter activity of the *protein kinase a catalytic subunit alpha* gene in transfection-reporter assays; KLF13, but not KLF9 repressed the *calmodulin 3* promoter. Forskolin activation of a cAMP-dependent promoter was reduced after forced expression of *Klf9* or *Klf13*, but was enhanced in *Klf* gene knockout cells. Forced expression of *Klf9* or *Klf13* blocked cAMP-dependent neurite outgrowth in HT22 cells, and axon growth in primary hippocampal neurons, while *Klf* gene knockout enhanced the effect of elevated cAMP. Taken together, our findings show that KLF9 and KLF13 inhibit neurite/axon growth in hippocampal neurons, in part, by inhibiting the cAMP signaling pathway.

## Introduction

Neurogenesis is a dynamic developmental process that involves the proliferation of neuronal precursors, then their migration, differentiation and elongation of projections. It is regulated by external cues and the expression of genes that promote axon growth and synaptogenesis (Tedeschi, 2012). Once axons have reached their targets and established functional synapses, a new genetic program is expressed that, together with external cues, maintains neural cell differentiation and inhibits axon growth/regeneration (Geoffroy et al., 2016; Hilton and Bradke, 2017). It is well known that the intracellular second messenger cyclic adenosine monophosphate (cAMP) plays a critical role in mediating the actions of extracellular signals on neuronal morphology and the establishment of neural circuits during development (Averaimo and Nicol, 2014), and that the activity of the cAMP signaling pathway declines in parallel with reduced regenerative capacity of mammalian neurons (Cai et al., 2001; Batty et al., 2017).

The developmental decline in cAMP production in the brain is coordinate with a reduction in the expression of enzymes that synthesize or degrade it (Cai et al., 2001). However, how this process is regulated is poorly understood. Recently, Krüppel-like factors (KLFs) have emerged as key players in regulating mammalian neuronal differentiation. They are zinc-finger transcription factors that bind to GC/GT rich sequences in DNA to regulate gene transcription (Pei and Grishin, 2013; Knoedler and Denver, 2014), and some KLFs have been shown to strongly restrict regenerative capacity of adult mammalian neurons (Moore et al., 2011). We have focused our studies on two members of the KLF subgroup 3, KLF9 and KLF13, which are encoded by paralogous genes that arose through a gene or genome duplication event in the vertebrate lineage (Lomberk and Urrutia, 2005). They, along with other members of the group 3 KLF subfamily (KLF10, KLF11, KLF14, and KLF16), share a common domain that binds the co-repressor protein Swi-independent 3a (Sin3a) (McConnell and Yang, 2010). These KLFs appear to work predominantly as transcriptional repressors, although they can also activate transcription depending on the cellular context (Knoedler and Denver, 2014; Bialkowska et al., 2017; Knoedler et al., 2017; Ávila-Mendoza et al., 2020).

The expression of *Klf9* in rodent brain increases during postnatal development, which is in accordance with its ability to first promote, then to maintain neuronal differentiation, and this expression pattern corresponds with a decrease in regenerative capacity (Shewan et al., 1995; Denver et al., 1999; Cayrou et al., 2002; Bonett et al., 2009; Apara et al., 2017; Tedeschi and Bradke, 2017). The developmental expression pattern of *Klf9* depends on the postnatal increase in thyroid hormone (T_3_), which directly regulates *Klf9* gene transcription via a T_3_ response element within an ultraconserved upstream superenhancer (the *Klf9* synergy module – KSM) (Denver et al., 1999; Denver and Williamson, 2009; Bagamasbad et al., 2015). Thyroid hormone plays critical roles in neural cell development and circuit formation (Bernal, 2007; Préau et al., 2015; Noda, 2018), and KLF9 has been shown to mediate T_3_ actions on neuron and oligodendrocyte differentiation (Cayrou et al., 2002; Avci et al., 2012; Dugas et al., 2012). The *Klf9* gene is also strongly induced by stress hormones (glucocorticoids) via two evolutionarily conserved glucocorticoid response elements located within and immediately upstream of the KSM (Bagamasbad et al., 2012). The developmental expression in the brain of the paralogous gene *Klf13* is currently unknown; it appears to be unaffected by T_3_, but is strongly induced by glucocorticoids in neuronal cells (Bagamasbad et al., 2019) and in cardiomyocytes via a glucocorticoid response element located in the first intron (Cruz-Topete et al., 2016). Glucocorticoids also have important roles in the central nervous system (Maggi et al., 2013; Joëls, 2018), and both KLFs may mediate stress hormone actions on neural cell development and function.

Our previous work investigating the molecular mechanisms of KLF9 and KLF13 actions in hippocampal neurons showed that they function predominantly as transcriptional repressors by associating in chromatin within proximal promoters of their target genes (Knoedler et al., 2017; Ávila-Mendoza et al., 2020). Furthermore, these paralogs have some compensatory and overlapping functions, such as cytoprotection and the regulation of the cellular circadian clock (Li et al., 2019; Ávila-Mendoza et al., 2020; Knoedler et al., 2020). However, they also have opposing actions, such as their effects on cell cycle progression (Knoedler et al., 2017; Ávila-Mendoza et al., 2020). While it is known that KLF9 and KFL13 inhibit axon growth of cortical neurons (Moore et al., 2009), the mechanisms that underlie these actions have not been fully elucidated. Our recent genome-wide analyses revealed that KLF9 and KLF13 may impact the dynamics of axon growth and regeneration by affecting several key cellular signaling pathways, including the cAMP and neurotrophin signaling pathways.

Here, we tested the hypothesis that KLF9 and KLF13 inhibit neurite/axon growth in mouse hippocampus-derived neurons by repressing the cAMP signaling pathway. We analyzed the effects of forced expression of *Klf9* or *Klf13* on cAMP pathway genes, KLF9 and KLF13 association in chromatin at the promoters of these genes, and the ability of these KLFs to directly regulate promoter activity. We also investigated if KLF9 and KLF13 can influence the overall activity of the cAMP signaling pathway in neurons using cAMP-dependent reporter assays. Lastly, we analyzed the effects of KLF9 and KLF13 on neurite outgrowth, and axon growth induced by cAMP pathway activation. Our findings support that KLF9 and KLF13 are capable of inhibiting process formation and growth in hippocampal neurons, in part by repressing cAMP pathway activity, and that KLF13 has a larger role than KLF9.

## Materials and Methods

### Animals

We purchased wild type (wild type; C57/B16 strain) mice from Jackson Laboratories, *Klf9*^–/–^ mice were kindly provided by Dr. Frank Simmen, and Klf13^–/–^ mice were provided by Dr. Raul Urrutia (Morita et al., 2003; Zhou et al., 2007). We re-derived the *Klf9*^–/–^ and *Klf13*^–/–^ mice into the C57/Bl6J strain (using the University of Michigan Transgenic Mouse Core) and backcrossed them for at least five generations prior to conducting experiments. Mice were kept on a 12L:12D photoperiod with food and water provided *ad libitum*, and were euthanized by rapid decapitation. All experimental procedures involving animals were conducted under an approved animal use protocol (PRO00008816) in accordance with the guidelines of the Institutional Animal Care and Use Committee at the University of Michigan.

### Bioinformatics Analysis

We analyzed data from our previously published studies (Knoedler et al., 2017; Ávila-Mendoza et al., 2020) in which we conducted RNA sequencing (RNA-seq) and chromatin-streptavidin precipitation sequencing (ChSP-seq) to identify KLF9 and KLF13 target genes in the mouse hippocampus-derived cell line HT22. We used DESeq2 to conduct differential expression analysis, and then analyzed differentially expressed genes in the context of the signaling pathways from the Kyoto Encyclopedia of Genes and Genomes (KEGG) database (Kanehisa and Goto, 2000; Kanehisa, 2002). One of the enriched pathways that we found relevant to the modulation of neuronal morphology is the cAMP signaling pathway. We therefore used iPathway Guide (Advaita Bioinformatics) to generate cAMP signaling pathway plots showing genes that were induced or repressed following forced expression of *Klf9* or *Klf13* (doxycycline [dox] induction for 8 h) (Knoedler et al., 2017; Ávila-Mendoza et al., 2020). We visualized the KLF9 and KLF13 ChSP-seq peaks that we identified previously using the Integrative Genome Viewer (IGV) (Robinson et al., 2011).

### Cell Culture and Transfection

We used the mouse hippocampus-derived cell line HT22, which has characteristics of adult hippocampal neurons (Morimoto and Koshland, 1990; Davis and Maher, 1994), and which we previously engineered to control the expression of a *Klf9* or a *Klf13* (*V5Klf13*) transgene under the control of doxycycline (Knoedler et al., 2017; Ávila-Mendoza et al., 2020). These cell lines are designated HT22-TR-3 (control cell line expressing the tetracycline repressor [TR]), HT22-TR/TO-*Klf9* and HT22-TR/TO-*V5Klf13*-1. We previously validated these platforms by showing that dox induction of the *Klf* transgenes corresponded with increases in functional proteins. We used a reporter assay as a proxy for KLF9 protein level in the cell since we were unable to detect it using Western blotting (Knoedler et al., 2017), and for KLF13 we showed time-dependent increases in V5KLF13 protein using Western blotting for the V5 tag (Ávila-Mendoza et al., 2020). We also used HT22 cells in which we inactivated the *Klf9*, *Klf13* or both genes using CRISPR/Cas9 genome editing (Knoedler et al., 2017, 2020; Ávila-Mendoza et al., 2020). These cell lines are designated HT22-*Klf9*-KO, HT22-*Klf13*-KO and HT22-*Klf9/13*-dKO (double KO). We cultured cells in high-glucose DMEM (Invitrogen) supplemented with 10% fetal bovine serum (Corning) and penicillin G (100 U/ml) plus streptomycin (100 μg/ml; Gibco) at 37°C under an atmosphere of 5% CO_2_. The culture medium for cells expressing the tet repressor (TR) was supplemented with 5 μg/ml blasticidin (Research Products International), while the medium for cells expressing *Klf9* or *V5Klf13* transgenes was supplemented with 100 μg/ml zeocin (InvivoGen).

To validate our RNA-seq data for KLF9 and KLF13 effects on cAMP signaling pathway genes, we forced expression of the *Klf* genes in the HT22-TR/TO-*Klf9* and HT22-TR/TO-*V5Klf13*-1 cell lines by treating them with dox. We plated cells at a density of 5 × 10^5^ cells in 6 well plates. Twenty hours after plating we treated cells with vehicle or dox (1 μg/ml) and eight hr later we harvested cells, extracted total RNA, synthesized cDNA and quantified gene expression by reverse transcriptase-quantitative polymerase chain reaction (RT-qPCR; described below).

We isolated primary cells from the hippocampus of WT, *Klf9*^–/–^ and *Klf13*^–/–^ PND1 mice as described previously (Ávila-Mendoza et al., 2020) following the protocol of Cazares et al. (2016), which generates cultures that are enriched for neurons. We plated cells onto 12 mm coverslips (pre-coated with 0.1 mg/ml 150–300 kDa Poly-L-Lysine; Sigma) at a density of 5 × 10^4^ and cultured them in 2 ml of complete Neurobasal medium (Gibco) supplemented with 2% B27 (Gibco) and 1X Glutamax (Gibco) at 37°C under an atmosphere of 5% CO_2_. Four hr later we added vehicle (0.05% DMSO) or 5 μM forskolin (FK; Sigma) and continued the cultures for 4 days before fixing cells for morphological analysis (described below). For transfection, we resuspended 1–3 × 10^6^ cells from WT PND1 mice in 100 μl of nucleofection buffer (P3 Primary Cell 4D-Nucleofector Kit; Lonza) and then added 2 μg of plasmid DNA: pTO-*Egfp* plus pCDNA4:TO (Invitrogen) empty vector (control) or pTO-*Egfp* plus pTO-*Klf9*, or pTO-*Egfp* plus pTO-*Klf13* at equimolar 1:1 ratio. We transfected cells using a Nucleofector (Lonza) with the EM110 program following the manufacturer’s instructions. We then added 2 ml of pre-warmed complete Neurobasal medium and plated transfected cells onto 12 mm coverslips at a density of 5 × 10^4^.

### RNA Extraction and RT-qPCR

We extracted total RNA from HT22 cells or mouse hippocampal tissue using the TRIzol Reagent (Invitrogen) following the manufacturer’s instructions. For each sample, we treated 1 μg total RNA with 20 IU of DNase I (Promega) for 30 min at 37°C, then synthesized cDNA using the High Capacity Reverse Transcription Kit with ribonuclease inhibitor (Applied Biosystems). We conducted quantitative real-time PCR using a StepOne Systems machine (Applied Biosystems) with qPCRBIO SyGreen Blue Mix Lo-Rox (PCRBiosystems). We designed oligonucleotide primers to span exon–exon boundaries where possible (Table 1) using the BLAST primer algorithm^1^. We generated standard curves by pooling cDNA samples and preparing serial dilutions for relative quantification, and normalized all genes to the geometric mean of the mRNA levels of the reference genes *TATA-box binding protein* (*Tbp*) and *peptidylprolyl isomerase A* (*Ppia*) (HT22 cells), or *glyceraldehyde-3-phosphate dehydrogenase* (*Gapdh*) and *Ppia* (hippocampal tissue), whose mRNAs were unaffected by the treatments.

**TABLE 1:** Oligonucleotides used for cloning, reverse transcriptase quantitative PCR (RT-qPCR), chromatin immunoprecipitation assays and genotyping.

	Sequences (5′ – 3′)	
	Forward	Reverse
**For cloning to create the vectors**		
pGL4.23-*Klf16* (promoter)	ATAGCTAGCCCTGTCCCAGTCTCAAAG	ATTAAGCTTAACCCTGCGCGAGAGTCTTC
pGL4.23-*Prkaca* (promoter)	ATAGCTAGCTAATGCACTTGGCTGGCGTCCG	CTCAAGCTTGGCCCCGGCGGTCATACATGC
pGL4.23-*Calm3* (promoter)	ATGCTAGCTCCATCTGCTGCTAAAACCG	TGAAGCTTCAGGTCGTGCTGCCCCCCGC
pGL4.23-*xcrfb* (promoter)	CATGCTAGCGGGCATAACAGCATATTA	GATATCTCTACAAACAGAAGTC
**For RT-qPCR**		
*Adcy6* mRNA	TTTGATTTTGGCCTGGCAGC	AGGAAGAGCACCACGTTAGC
*Calm3* mRNA	GGCTATATTAGCGCTGCCGA	CTCCATCAATGTCGGCCTCT
*Gadph* mRNA	TGTGTCCGTCGTGGATCTGA	CTTCACCACCTTCTTGATGTCACT
*Klf9* mRNA	TCCTCCCATCTTAAAGCCCAT	CCGAGCGCGAGAACTTT
*Klf13* mRNA	CGAGCCTGGCCTCAGACAAA	CTTTCTCGCAGCCCGCGTA
*Klf16* mRNA	CTGTCCCTTCCATGGCTG	ATCAGAACTGGCGAACTTC
*Ppia* mRNA	GGTTCCTCCTTTCACAGAAT	AATTTCTCTCCGTAGATGGAC
*Prkaca* mRNA	GCGAGCAGGAGAGCGTGAAA	CACCAGCATCACTCGCCCAA
*Rap1a* mRNA	ATGGCCAAGGGTTTGCACTA	TCTGGCCTTGTTCTTTGCCA
*Rapgef3* mRNA	GGTGAAGGTCAATTCTGCCGGT	CACCTGGTGGATCCTGTTGAAGA
*Tbp* mRNA	CCGTGAATCTTGGCTGTAAACTTG	GTTGTCCGTGGCTCTCTTATTCTC
*Tiam1* mRNA	AGTCGCACTGTCTTTCCGAG	GGCGAGTAGCTTGAGTTGGT
*Vav2* mRNA	GCCCATGAAAATGGGCATGA	CGCAAGGGACCCATGTAGTT
**For ChIP-qPCR**		
*Klf16* intron	ACTAAACTCCACCCCACAAC	TCTTTCAAACACTCCCTCGC
KLF13 peak at *Klf16* promoter	GTACGCACTACCCTCACCAG	GGTGGGCGTAACTCTCAAAG
KLF13 peak at *Prkaca* promoter	CAGCACGCCCTCAGTTCTGG	GTCCGCTTTGGTTTGCTCGC
KLF13 peak at *Rap1a* promoter	GAAGACCGGAATCACACCGTGG	CAGCGTCGCTCTCGACTCTCT
KLF13 peak at *Calm3* promoter	CTTCCACAGAGCCCAGCGAAT	CGCCGAGAGCGAAAGTAGTCC
KLF13 peak at *Adcy6* promoter	CTCCCTTGTTGTCCAGCGCA	CCTTTAAGGCGGGGAGTCCG
KLF9 peak at *Rapgef3* promoter	TCGGGTAGGGACTCCACTAG	AGGCACGAGCTTTACGGTAG
**To genotype Klf13-null mice**		
*Klf9* allele	AGCGCGAGGTGACCAAGGAA	CGGGCTGTGGGAAGGACTCG
*Klf9*-NEO cassette	ATGAACTGCAGGACGAGGCAGCG	GGCGATAGAAGGCGATGCGCT
*Klf13* allele	CTCGGTAATGTCCCGCCCATA	AGAGTCGGCCTGTCTTAGGGA
*Klf13*-NEO cassette	CTCGGTAATGTCCCGCCCATA	AAGCCGGTCTTGTCAATCAGGATGATCTGGACG

### Chromatin Extraction and Immunoprecipitation

We extracted chromatin from cells and hippocampus, and conducted ChIP assays as described previously (Denver and Williamson, 2009; Knoedler et al., 2017; Ávila-Mendoza et al., 2020). Briefly, we treated HT22-TR/TO-*Klf9* and HT22-TR/TO-*V5Klf13*-1 cells with vehicle or dox (1 μg/ml) for 16 h, then extracted and sheared the chromatin to 200–600 bp using an M220 Focused-Ultrasonicator (Covaris) for 20 min with a 5% duty factor. For each ChIP reaction we used 50–100 μg of chromatin and incubated it with 5 μg purified goat anti-KLF9 IgG (Santa Cruz Biosciences) or with 1 μg of our custom affinity-purified anti-KLF13 IgG (Ávila-Mendoza et al., 2020). As a control we precipitated chromatin with 5 μg of normal goat IgG (Santa Cruz Biosciences) or with 1 μg of IgG purified from rabbit pre-immune serum. We conducted relative quantification of the immunoprecipitated DNA using SYBR Green qPCR with standard curves prepared from serial dilutions of genomic DNA isolated from HT22 cells using the DNeasy Blood & tissue Kit (Qiagen). Precipitated samples were normalized as a percentage of the corresponding input sample. We also conducted ChIP assays on hippocampal tissue isolated from PND1 and PND30 WT mice, or PND30 *Klf9*^–/–^ or *Klf13*^–/–^ mice (negative controls). We normalized the ChIP KLF9 or KLF13 quantities to the normal IgG quantities.

### Dual Luciferase Promoter-Reporter Assays

We conducted promoter-reporter assays using the HT22-TR/TO-*Klf9* and HT22-TR/TO-*V5Klf13* cell lines to investigate if KLF9 or KLF13 can directly regulate transcriptional activity of the promoters of cAMP signaling pathway genes. We amplified genomic regions that corresponded to KLF13 peaks (identified by ChSP-seq) (Ávila-Mendoza et al., 2020) located within the promoters of *Klf16* (2200 bp), *Prkaca* (1300 bp) and *Calm3* (700 bp), and we directionally cloned the DNAs into the pGL4.23 vector (Promega) at the *Nhe*I and *Hin*dIII sites. We plated cells in 24 well plates at a density of 5 × 10^4^ cells/well in growth medium (DMEM with 10% FBS). The following day, we replaced the medium with DMEM plus 1% FBS containing vehicle or dox (1 μg/ml), then we co-transfected cells with 200 ng of the pGL4.23 plasmids plus 10 ng of the promoter-less pRenilla vector using Fugene6 (Invitrogen). Twenty hour later we harvested cells and conducted Dual Luciferase Reporter Assay (Promega) following the manufacturer’s instructions. We normalized the firefly luciferase to the Renilla luciferase values, and we present the data as relative luciferase activity (RLA).

We also conducted transfection reporter assays using a cAMP responsive promoter-reporter vector to monitor activity of the cAMP signaling pathway in cells. For these experiments we used the *Klf9* and *V5Klf13* dox-inducible, and the *Klf9*-KO, *Klf13*-KO and double KO HT22 cell lines. We transfected cells with the pGLxCRF vector, which contains a 431 bp fragment of the gene promoter of the *Xenopus laevis corticotropin releasing factor b* (*crfb*) gene which has a functional cAMP response element (CRE) driving firefly luciferase (Yao et al., 2007). We co-transfected cells with the promoter-less pRenilla plasmid (Invitrogen) to normalize luciferase activity. Cell cultures were prepared and transfected with 200 ng of pGLxCRF vector plus 10 ng pRenilla, as described above. We treated cells with vehicle (0.1% DMSO) or 25 μM forskolin (FK) plus 250 μM 3-isobutyl-1methylxanthine (IBMX) for 4 h before harvesting cells for dual luciferase assay. We treated the TR/TO cells with vehicle or dox (1 μg/ml) 4 hr before initiating FK + IBMX treatment. All transfection reporter assays were repeated at least two times with four replicates/treatment.

### Neurite Outgrowth Assay in HT22 Cells

We induced neurite outgrowth in HT22 cells by activating the cAMP signaling pathway with FK + IBMX. We used the *Klf9* and *V5Klf13* dox-inducible, and the *Klf9*-KO, *Klf13*-KO and double KO HT22 cell lines. We plated cells at a density of 1.5 × 10^4^ cells/well in 12 well plates. The following day we replaced growth medium with DMEM plus 1% FBS; the TR/TO cells were treated with vehicle or dox (1 μg/ml) for 4 h before initiating FK + IBMX treatment. Cells then received 0.1% DMSO vehicle or 25 μM FK + 250 μM IBMX for 16 h before washing with PBS and fixing with 4% paraformaldehyde plus 4% sucrose in PBS (pH 7.4). We acquired images under bright field illumination with an Olympus IX81 inverted microscope using a 10 X objective. For each treatment we imaged at least 10 random fields. We analyzed a minimum of 100 neurons per treatment from three independent experiments. We estimated neurite outgrowth as the ratio between the longest neurite and the soma diameter as described by Inda et al. (2017) using the Simple Neurite Tracer plugin (Frangi et al., 1998) into the Fiji platform (Schindelin et al., 2012).

### Axon Growth Assay in Primary Hippocampal Neurons

We analyzed the impact of gain or loss of function of KLF9 or KLF13 on cAMP-induced axon growth in primary hippocampal neurons. We transfected primary cells isolated from wild type PND1 mice with pTO-*Egfp*, pTO-*Klf9* or pTO-*Klf13* then cultured them for 4 days before conducting immunocytochemistry (ICC). We also isolated primary cells from wild type, *Klf9*^–/–^ or *Klf13*^–/–^ mice and treated them with vehicle (0.05% DMSO) or FK (5 μM) for 4 days before ICC. Unlike with HT22 cells, we used only FK in primary neurons since co-treatment with IBMX resulted in cell death.

For ICC we first fixed cells with 4% paraformaldehyde plus 4% sucrose in PBS (pH 7.4) for 15 min, then permeabilized them in 0.2% Triton X-100 for 10 min, washed two times with PBS and incubated in blocking buffer (5% BSA in PBS) for 1 h. We then incubated cover slips with a mouse monoclonal anti β-tubulin III primary antibody (1:500, Millipore, MAB1637) in 2% BSA at 4°C for 16 h followed by three washes with PBS containing 0.05% Triton X-100. Immune complexes were revealed by incubation with a Cy5-conjugated goat anti mouse IgG secondary antibody (1:1000, Jackson ImmunoResearch) for 2 h. We then washed the coverslips three times with PBS and mounted them with Prolong Gold Antifade mountant medium (Invitrogen). We acquired images with an Olympus IX81 inverted fluorescence microscope using a 10 X objective lens. We measured and analyzed the longest β-tubulin-stained projection.

### Statistical Analysis

All data are expressed as the mean ± standard error of the mean (SEM). We analyzed data by Student’s independent *t*-test or by one-way analysis of variance (ANOVA) followed by Tukey’s multiple comparison test using Prism8 (GraphPad). Derived values were tested for homogeneity of variance using the Brown–Forsythe test, and when appropriate the data were Log_10_-transformed before analysis. A *p*-value < 0.05 was considered statistically significant.

## Results

### KLF9 and KLF13 Regulate Several cAMP Signaling Pathway Genes in HT22 Cells

**FIGURE 1:**
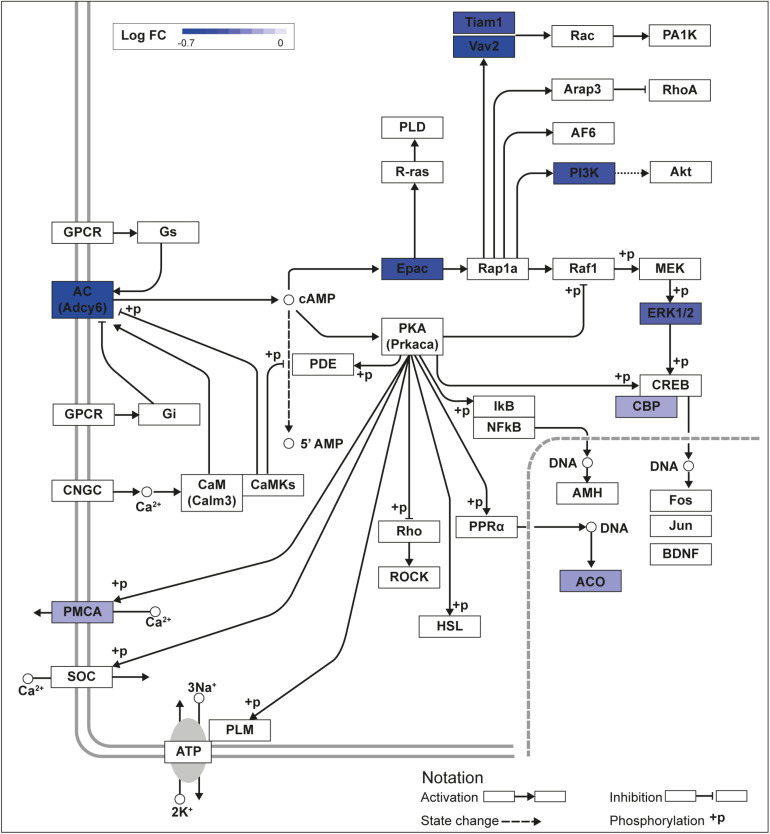
KLF9 regulates cAMP signaling pathway genes in HT22 cells. We previously conducted an RNA sequencing experiment in the HT22-TR/TO-*Klf9* cell line treated with or without doxycycline for 8 h to induce the *Klf9* transgene (Knoedler et al., 2017). We analyzed differentially regulated genes in the context of the Kyoto Encyclopedia Genes and Genomes signaling pathways. Shown is the core of the cAMP signaling pathway (KEGG:04024) overlayed with differential gene expression values for each gene with a *p*-value < 0.05. The box shading for each gene represents the direction of change in mRNA levels (Log fold change – Log FC shown in the legend); i.e., boxes with darkest shading indicate the largest decreases in mRNA levels.

**FIGURE 2:**
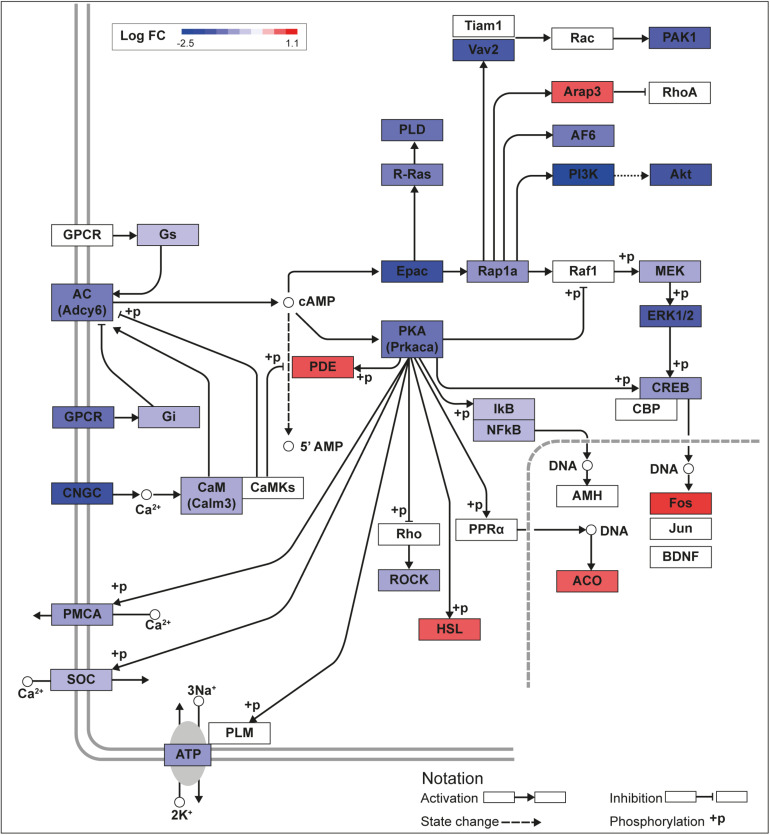
KLF13 regulates cAMP signaling pathway genes in HT22 cells. We previously conducted an RNA sequencing analysis in the HT22-TR/TO-*V5Klf13*-1 cell line treated with or without doxycycline for 8 h to induce the *V5Klf13* transgene (Ávila-Mendoza et al., 2020). We analyzed differentially regulated genes in the context of the Kyoto Encyclopedia Genes and Genomes signaling pathways. Shown is the core of the cAMP signaling pathway (KEGG:04024) overlayed with differential gene expression values for each gene with a *p*-value < 0.05. The color of the box for each gene represents the direction of change in mRNA levels (Log fold change – Log FC shown in the legend); i.e., boxes with dark blue indicate the largest decreases, while boxes with dark red indicate the largest increases in mRNA levels.

**FIGURE 3:**
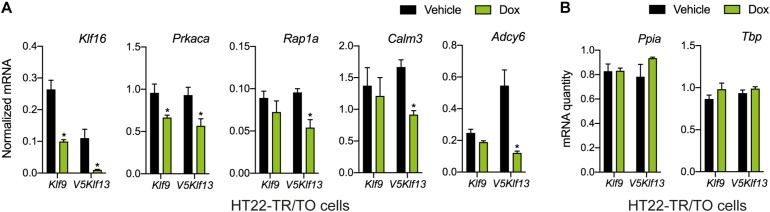
Targeted analysis of cAMP signaling pathway gene repression by KLF9 and KLF13 in HT22 cells. We treated the HT22-TR/TO-*Klf9* and HT22-TR/TO-*V5Klf13*-1 cell lines with vehicle or doxycycline (dox; 1 μg/ml) for 8 h, then harvested cells, isolated RNA and conducted RT-qPCR. Bars represent the mean ± SEM. **(A)** Changes in *Klf16* mRNA levels served as a positive control. Forced expression of *Klf9* reduced the mRNA levels of *Klf16* (*p* = 0.0004) and *Prkaca* (*p* = 0.02) while forced expression of *V5Klf13* reduced the mRNA levels of *Klf16* (*p* = 0.0037), *Prkaca* (*p* = 0.026), *Rap1a* (*p* = 0.007), *Calm3* (*p* = 0.001), and *Adcy6* (*p* = 0.001); *n* = 4/treatment; Student’s independent *t*-test. Asterisks indicate statistically significant differences within a cell line with *p* < 0.05. **(B)** We normalized target gene mRNA levels shown in **(A)** to the geometric mean of the mRNA levels of the reference genes *Tbp* and *Ppia*, which were unaffected by dox treatment.

We recently published RNA-seq transcriptome analyses for KLF9 and KLF13 in HT22 cells (Knoedler et al., 2017; Ávila-Mendoza et al., 2020). Forced expression of *Klf9* or *V5Klf13* impacted several cellular signaling pathways, including the cAMP signaling pathway; KLF9 reduced the mRNA levels of 10 genes associated with this pathway (Figure 1 and Table 2), while KLF13 affected 45 genes (33 repressed, 12 induced; Figure 2 and Table 2). We chose seven genes based on their central positions in the pathway for targeted analysis by RT-qPCR after forced expression of *Klf9* or *V5Klf13* for 8 h. As expected, both KLFs strongly reduced *Klf16* mRNA levels (used as a positive control; Knoedler et al., 2017; Ávila-Mendoza et al., 2020). The six genes that we tested that were previously found by RNA-seq to be repressed by V5KLF13 (*protein kinase a catalytic subunit alpha – Prkaca, Ras-related protein 1a* – *Rap1a, calmodulin 3 – Calm3, adenyl cyclase 6* – *Adcy6*, *Rap guanine nucleotide exchange factor 3 – Rapgef3* [also known as *EPAC1*] and *VAV guanine nucleotide exchange factor-2* – *Vav2*) were all validated by targeted RTqPCR (Figure 3A and Supplementary Figure 1A). Of the four genes that we tested that we previously found by RNA-seq to be repressed by KLF9 (*Adcy6*, *Rapgef3*, *TIAM Rac1 associated GEF-1* – *Tiam1*, and *Vav2*) only *Rapgef3* and *Tiam1* validated by targeted RTqPCR; the mean mRNA levels of *Adcy6* and *Vav2* were lower after doxycycline (dox) treatment compared with vehicle treated controls, but this was not statistically significant (Figure 3A and Supplementary Figure 1A). The *Prkaca* mRNA level was not affected by KLF9 in the RNA-seq dataset, but was found to be reduced by targeted RTqPCR (Figure 3A). There were no effects of forced expression of *Klf9* or *V5Klf13* on the mRNA levels of the reference genes *Tbp* or *Ppia* (Figure 3B).

**TABLE 2:** cAMP pathway genes regulated by forced expression of *Klf9* or *V5Klf13*.

Gene	KLF9		KLF13	
	Log FC	Adj *p*	Log FC	Adj *p*
*Acox1*	−0.29	0.001619		
*Acox3*			0.68	0.000001
*Adcy3*			0.95	0.000932
*Adcy6*	−0.37	0.00262	−1.21	0.000001
*Adcy7*	−0.77	0.000448		
*Adrb2*			0.71	0.037008
*Afdn*			−1.22	0.000001
*Akt1*			−0.73	0.000001
*Akt2*			−1.07	0.000001
*Akt3*			−1.7	0.000001
*Arap3*			0.7	0.007717
*Atp1a3*			−0.95	0.000001
*Atp2b1*	−0.26	0.000817	−0.9	0.000001
*Calm2*			−0.62	0.000001
*Calm3*			−0.77	0.000001
*Cngb1*			−1.82	0.000001
*Creb1*			−0.64	0.049385
*Creb3*			0.71	0.000001
*Creb3l1*			−0.96	0.000001
*Crebbp*	−0.26	0.001503		
*Fos*			0.85	0.002864
*Gabbr1*			−1.33	0.000001
*Ghrl*			1.06	0.012682
*Gli1*			−0.93	0.028606
*Gnai1*			0.69	0.028579
*Gnai2*			−0.59	0.000001
*Gnai3*			−0.73	0.000001
*Gnas*			−0.61	0.000001
*Lipe*			0.69	0.000001
*Map2k2*			−0.81	0.000001
*Mapk3*	−0.45	0.00547	−1.54	0.000001
*Nfkbia*			−0.6	0.000001
*Orai1*			−0.68	0.028441
*Pak1*			−1.5	0.000001
*Pde4b*			0.73	0.000058
*Pik3cd*	−0.56	0.00096	−2.46	0.000001
*Pik3r2*			−1.56	0.000001
*Pik3r3*			1.03	0.000001
*Pik3r5*			−2.29	0.000001
*Pld2*			−1.31	0.000001
*Prkaca*			−1.3	0.000001
*Prkacb*			−0.73	0.000001
*Rap1a*			−0.98	0.000001
*Rapgef3*	−0.6	0.000046	−1.86	0.000001
*Rela*			−0.66	0.000001
*Rock2*			−0.84	0.000001
*Rras*			−1.17	0.000001
*Tiam1*	−0.55	0.000614		
*Vav2*	−0.68	0.000001	−1.6	0.000001

### KLF9 and KLF13 Associate in Chromatin at Promoters of cAMP Signaling Pathway Genes in HT22 Cells

**FIGURE 4:**
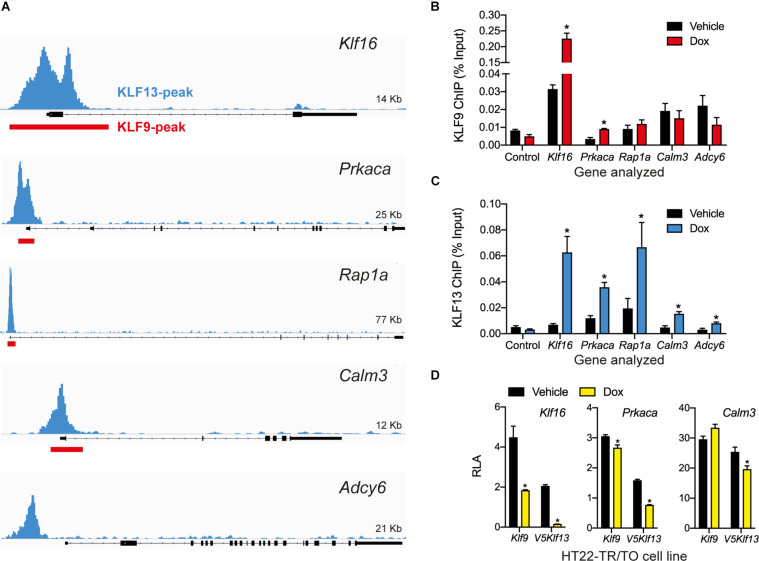
KLF9 and KLF13 associate in chromatin at promoters of cAMP signaling pathway genes in HT22 cells. **(A)** Screenshots of genome traces from the Integrative Genome Viewer (IGV) showing the locations of KLF13 chromatin-streptavidin precipitation sequencing (ChSP-seq) peaks at genes in the cAMP signaling pathway (Ávila-Mendoza et al., 2020). The gene structures are shown below the genome traces, with lines and black filled bars representing introns and exons, respectively. The gene orientations are 5′ 3′, and all peaks are within the proximal promoter regions of the genes shown. Red boxes indicate locations of KLF9 peaks identified previously by ChSP-seq (Knoedler et al., 2017). **(B,C)** We conducted targeted ChIP-qPCR assays in HT22 cells to validate the KLF9 and KLF13 ChSP-seq peaks at cAMP signaling pathway genes. We treated HT22-TR/TO-*Klf9* and HT22-TR/TO-*V5Klf13*-1 cells with vehicle or doxycycline (dox; 1 μg/ml) for 16 h, then harvested cells and isolated chromatin for ChIP assays. The KLF9 and KLF13 ChIP qPCR data are expressed as a percentage of the input. We analyzed the *Klf16* intron, which did not have KLF9 or KLF13 ChSP peaks, as a negative control region (Control). Bars represent the mean ± SEM (*n* = 4/treatment), and we used Student’s independent *t*-test to compare the ChIP values between vehicle and dox treatment within a cell line (asterisks indicate *p* < 0.05). **(B)** KLF9 ChIP qPCR assay conducted on chromatin isolated from HT22-TR/TO-*Klf9* cells (*Klf16: p* < 0.0001; *Prkaca: p* = 0.0024). **(C)** KLF13 ChIP qPCR assay conducted on chromatin isolated from HT22-TR/TO-*V5Klf13*-1 cells (*Klf16: p* = 0.0006; *Prkaca: p* = 0.0044; *Rap1a: p* = 0.032; *Calm3: p* = 0.027; *Adcy6: p* = 0.048). **(D)** KLF9 and KLF13 regulate promoter activity in transfection promoter-reporter assays conducted in HT22 cells. We co-transfected HT22-TR/TO-*Klf9* or HT22-TR/TO-*V5Klf13*-1 cells with pRenilla plus firefly luciferase reporter vectors containing genomic DNA fragments corresponding to KLF9 and KLF13 ChSP peaks [shown in **(A)**] within the promoters of the *Klf16* (positive control), *Prkaca* and *Calm3* genes. Twenty hr after transfection we treated cells with vehicle or dox (1 μg/ml) for 16 h, then harvested cells and conducted dual luciferase reporter assay. The relative luciferase activity (RLA) represents firefly luciferase normalized to the *Renilla* luciferase values. Forced expression of *Klf9* or *V5Klf13* repressed transcriptional activity of the *Klf16* and *Prkaca* promoters (HT22-TR/TO-*Klf9*: *Klf16, p* = 0.0004; *Prkaca, p* = 0.018; HT22-TR/TO-*V5Klf13*-1: *Klf16, p* < 0.0001; *Prkaca, p* < 0.0001; *n* = 4/treatment; Student’s independent *t*-test); only forced expression of *V5Klf13* repressed transcriptional activity of the *Calm3* promoter (*Calm3, p* < 0.013; *n* = 4/treatment). Asterisks indicate statistically significant differences within a cell line with *p* < 0.05.

Our previous genome-wide analyses using ChSP-seq in HT22 cells (Knoedler et al., 2017; Ávila-Mendoza et al., 2020) found that KLF9 and KLF13 exhibit overlapping association in chromatin at genomic regions within 1 kb of the transcription start sites of *Klf16* (control), *Prkaca, Rap1a*, and *Calm3*; KLF13 but not KLF9 associated in chromatin at *Adcy6* (Figure 4A). Targeted ChIP-qPCR assays for KLF9 and KLF13 on chromatin isolated from HT22-TR/TO-*Klf9* or TR/TO-*V5Klf13*-1 cells treated with dox for 16 h caused robust increases in the KLF9 (7.1 fold) and KLF13 (9.8 fold) ChIP signals at the *Klf16* promoter (which served as a positive control); there was no change at the *Klf16* intron, which served as a negative control (Figures 4B,C). We saw a 2.7 fold increase in the KLF9 ChIP signal at the *Prkaca* promoter after dox treatment, but not at the promoters of the other three genes (Figure 4B). By contrast, we found statistically significant increases in the KLF13 ChIP signal after dox treatment at the promoters of each of the four genes tested: *Prkaca* (3 fold), *Rap1a* (3.4 fold), *Calm3* (3.3 fold) and *Adcy6* (2.6 fold). Of the other three genes analyzed by targeted RTqPCR (*Rapgef3, Tiam1* and *Vav2*), our ChSP-seq analyses (Knoedler et al., 2017; Ávila-Mendoza et al., 2020) showed that *Rapgef3* has a KLF9 but not a KLF13 peak at its promoter (although there are strong KLF13 peaks at adjacent genes; see Supplementary Figure 1B), *Vav2* has peaks for both KLFs, but *Tiam1* has no peaks for either KLF. Using the genomic region covered by the KLF9 peak at the promoter of the *Rapgef3* gene as the target sequence for ChIP-qPCR, we found that the KLF9 and KLF13 ChIP signals were enriched here after dox treatment by 2.1 and 2.3 fold, respectively (Supplementary Figure 1C).

To investigate if KLF9 and KLF13 can directly regulate gene promoter activity we conducted transfection-reporter assays in the dox-inducible HT22 cell lines. Forced expression of *Klf9* or *V5Klf13* caused strong reductions in RLA (60 and 92%, respectively) in cells transfected with the positive control reporter vector pGL4.23-*Klf16* (Figure 4D) (Knoedler et al., 2017; Ávila-Mendoza et al., 2020). Forced expression of *Klf9* or *V5Klf13* reduced RLA in cells transfected with pGL4.23-*Prkaca* by 12.6 and 52%, respectively. Forced expression of *V5Klf13* reduced RLA (by 16.3%) in cells transfected with pGL4.23-*Calm3*, but expression of *Klf9* had no effect.

### KLF9 and KLF13 Inhibit cAMP Signaling in HT22 Cells

**FIGURE 5:**
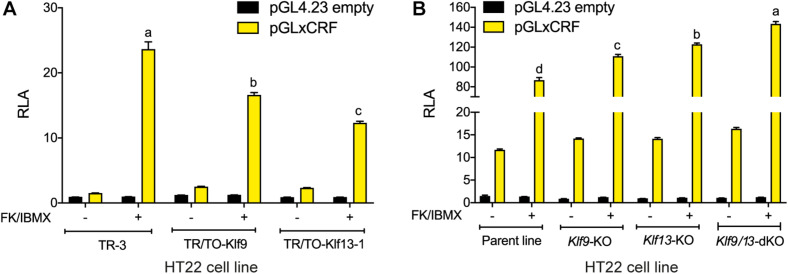
KLF9 and KLF13 repress cAMP signaling pathway activity in HT22 cells. **(A)** We co-transfected HT22-TR/TO-*Klf9* and HT22-TR/TO-*V5Klf13*-1 cells with pRenilla plus a firefly luciferase reporter vector containing the proximal promoter of the *Xenopus laevis corticotropin releasing factor b* (*crfb*) gene (pGLxCRF), which contains a functional cAMP response element (CRE) (Yao et al., 2007). This reporter is activated by phosphorylated CRE binding protein (CREB), and therefore reflects the activity of the cAMP signaling pathway in cells. Control cells were transfected with pGL4.23 empty vector. Twenty hour after transfection we treated cells with doxycycline (dox; 1 μg/ml) to induce expression of the *Klf9* or *V5Klf13* transgenes, and 4 hour later we added vehicle or FK + IBMX to elevate intracellular cAMP. Eight hr after initiating dox treatment we harvested cells and analyzed reporter activity using dual luciferase assay. Bars represent the mean ± SEM. Forced expression of *Klf9* or *V5Klf13* reduced the relative luciferase activity (RLA) induced by FK + IBMX treatment [*F*_(2,9)_ = 86.06, *p* < 0.0001; one-way ANOVA followed by Tukey’s *post hoc* test; *n* = 4/treatment]. Letters indicate statistically significant differences (*p* < 0.05) between cell lines treated with FK + IBMX. **(B)** We co-transfected HT22 parental and *Klf* knockout (KO) cell lines (HT22-*Klf9*-KO, HT22-*Klf13*-KO, and HT22-*Klf9/13*-double KO) as described above, then we treated them with FK + IBMX for 4 h before harvest and analysis by dual luciferase assay. Bars represent the mean ± SEM. The RLA induced by FK + IBMX was greater in each of the KO cell lines compared to the parental cell line, and was greatest in the double KO cells [*F*_(3,12)_ = 89.39, *p* < 0.0001; one-way ANOVA followed by Tukey’s *post hoc* test; *n* = 4/treatment]. Letters indicate statistically significant differences (*p* < 0.05) between cell lines treated with FK + IBMX.

Our findings support that KLF9 and KLF13 can negatively regulate components of the cAMP signaling pathway in neurons, with KLF13 having a larger effect on this pathway than KLF9. Here we tested if this negative regulation is reflected in a transcriptional readout of cAMP activity. We transfected cells with the pGxCRF vector, which functions as a sensor of the activity of the cAMP signaling pathway (Yao et al., 2007). Treatment with FK + IBMX for 4 h strongly increased RLA in HT22-TR-3 cells transfected with pGxCRF (15.8 fold; Figure 5A). This reporter activation was significantly reduced in HT22-TR/TO-*Klf9* cells (by 30%) and in HT22-TR/TO-V5*Klf13*-1 cells (by 48%) that were treated with dox for 4 h before, and also during the 4 h FK + IBMX treatment. Conversely, we found that the RLA response to FK + IBMX was augmented in HT22-*Klf9*-KO (27%), *Klf13*-KO (41%) and *Klf9/13*-double KO cells (64%) (Figure 5B; *Klf9/13* double-KO > *Klf13*-KO > *Klf9-*KO). The RLA of cells transfected with the empty pGL4.23 vector was unaffected by the treatments.

### *Klf9* and *Klf13* mRNAs Increase, and KLF13 Associates in Chromatin at the *Prkaca* Gene Promoter in Hippocampus During Postnatal Development

**FIGURE 6:**
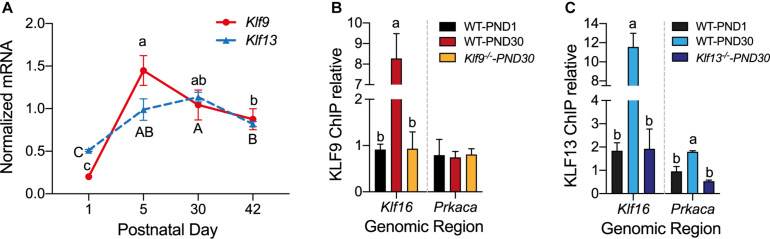
The mRNA levels for *Klf9* and *Klf13* increase, and KLF13, but not KLF9, associates in chromatin at the promoter region of *Prkaca* during postnatal development of the hippocampus. **(A)** Changes in *Klf9* and *Klf13* mRNA levels in mouse hippocampus during postnatal development. We collected the hippocampal region from mice at postnatal day (PND) 1, 15, 30, and 42, and we quantified *Klf9* and *Klf13* mRNAs by RT-qPCR. We normalized the *Klf* mRNA levels to the geometric means of the mRNA levels of the reference genes *Gadph* and *Ppia.* The mean mRNA levels of both *Klf* genes peaked at PND15, remained elevated at PND30, and declined at PND42. Points represent the mean ± SEM [*Klf9*: *F*_(3,26)_ = 55.84, *p* < 0.0001; *Klf13*: *F*_(3,12)_ = 24.99, *p* < 0.0001; one-way ANOVA followed by Tukey’s *post hoc* test; *n* = 4/treatment]. Means with the same letter are not significantly different. **(B,C)** We analyzed KLF9 and KLF13 occupancy in chromatin at the promoter regions of the *Klf16* and *Prkaca* genes in wild type (WT) mouse hippocampus at PND1 and PND30, and PND30 *Klf9*^–/–^ and *Klf13*^–/–^ mice using ChIP qPCR. We normalized the KLF9 and KLF13 ChIP values to the signal obtained with IgGs purified from normal goat (KLF9) or normal rabbit (KLF13) serum. Bars represent the mean ± SEM (*n* = 4/treatment). Means with the same letter within a gene analyzed are not significantly different (*p* < 0.05; one-way ANOVA followed by Tukey’s *post hoc* test.). **(B)** The KLF9 ChIP signal at the *Klf16* gene promoter was greater in WT PND30 mice compared to PND1, and also compared to *Klf9*^–/–^ PND30 mice (which was not different from PND1 WT mice) [*F*_(2,9)_ = 28.80, *p* = 0.0001]. The mean KLF9 ChIP signal at the *Prkaca* gene promoter was at background level (the level of the *Klf9*^–/–^ PND30 mice) in WT PND1 and PND30 mice. **(C)** The KLF13 ChIP signal at the *Klf16* gene promoter was greater in WT PND30 mice compared to PND1, and also compared to *Klf13*^–/–^ PND30 mice [which was not different from WT PND1 mice; *F*_(2,10)_ = 36.01, *p* < 0.0001]. The mean KLF13 ChIP signal at the *Prkaca* gene promoter in WT PND30 mice was greater than the level in WT PND1 and *Klf13*^–/–^ PND30 mice [*F*_(2,7)_ = 14.89, *p* = 0.003].

Analysis of the mRNA levels of *Klf9* and *Klf13* by RT-qPCR in the hippocampus of wild type mice at four postnatal ages (PND1, 15, 30, and 42) showed that expression of both genes was low at PND1, then increased 5.5 fold (*Klf9*) and 2 fold (*Klf13*) at PND15, remained elevated at PND30, then declined at PND42 (Figure 6A).

To test if changes in the mRNA levels result in changes in KLF association in chromatin *in vivo* we conducted ChIP assays for KLF9 and KLF13 using chromatin isolated from the hippocampus of wild type mice at PND1 and PND30. For negative controls we analyzed chromatin isolated from the hippocampus of *Klf9*^–/–^ and *Klf13*^–/–^ mice at PND30; these ChIP signals represent the background levels in the assay. The ChIP signal at the *Klf16* locus was enriched by 8.8 fold (KLF9) and 6 fold (KLF13) compared to their respective signals in PND1 wild type, and PND30 *Klf9*^–/–^ or *Klf13*^–/–^ mice (Figures 6B,C). The ChIP signal for KLF13 at the *Prkaca* promoter was increased by 3.3 fold above the background level in wild type PND30 mice (Figure 6C). The KLF9 ChIP signal at this promoter was not different from background in wild type mice at either of the two ages (Figure 6B). We also analyzed KLF9 and KLF13 association in chromatin at the promoters of *Rap1a* and *Calm3* and found that the ChIP signals were similar to background in wild type mice at both ages (data not shown).

### KLF9 and KLF13 Inhibit cAMP-Stimulated Neurite Outgrowth in HT22 Cells

**FIGURE 7:**
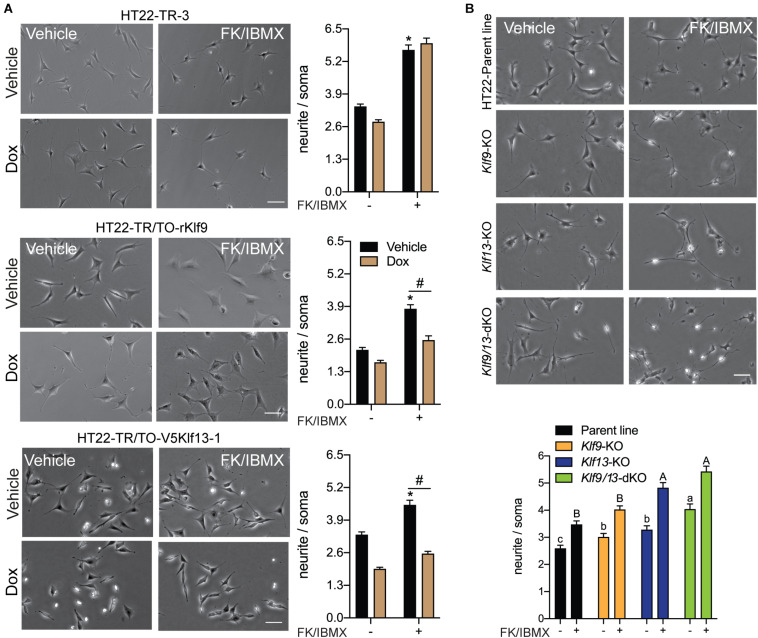
Forced expression of *Klf9* or *V5Klf13* block, while their deficiency enhances cAMP-dependent neurite-outgrowth in HT22 cells. We analyzed the effects of forced expression of *Klf9* or *V5Klf13* on cAMP-dependent neurite-outgrowth, or the effects of inactivation of the *Klf9* or *Klf13* genes using CRISPR/Cas9 genome editing on neurite outgrowth in HT22 cells. We estimated the length of neurites by calculating the ratio of the length of the longest neurite to the width of the soma of each cell analyzed. Bars represent the mean ± SEM (we analyzed > 100 cells/treatment from three independent experiments). **(A)** We treated HT22-TR-3 (control), TR/TO-*Klf9* and TR/TO-*V5Klf13*-1 cells with vehicle or doxycycline (dox; 1 μg/ml) for 4 h, then added forskolin (FK; 25 μM) plus IBMX (250 μM) for 16 h before fixing cells for morphometric analysis. Shown are representative micrographs with graphs depicting quantification of neurites to the right. Treatment with FK + IBMX induced neurite outgrowth in each of the three cell lines; asterisks indicate statistically significant differences (*p* < 0.0001; Student’s independent *t*-test) between cells receiving vehicle (no dox) treated ± FK + IBMX. Treatment with dox had no effect on the response to FK + IBMX in HT22-TR-3 cells, but dox treatment completely blocked the response in HT22-TR/TO-*Klf9* and HT22-TR/TO-*V5Klf13*-1 cells. Hashtags indicate statistically significant differences (*p* < 0.05; Student’s independent *t*-test) between vehicle and dox-treated cells that received FK + IBMX. **(B)** We treated HT22 *Klf9*-knockout (KO), *Klf13*-KO and *Klf9/13*-double-KO cells with vehicle or FK (25 μM) + IBMX (250 μM) for 16 h before fixing cells for morphometric analysis. Shown are representative micrographs with a graph depicting quantification of neurites at the bottom. Inactivation of *Klf9*, *Klf13* or both genes increased the baseline neurite length compared with the HT22 parent cell line [*F*_(3,514)_ = 15.63, *p* < 0.0001; one-way ANOVA followed by Tukey’s *post hoc* test]. Treatment with FK + IBMX increased neurite length in all four cell lines (*p* < 0.05, Student’s independent *t*-test), but neurite length was greater in the *Klf13*-KO and *Klf9/13*-double KO cells compared with the *Klf9*-KO and parental cell lines [*F*_(3,589)_ = 24.13, *p* < 0.0001]. Means with the same letter are not significantly different (lowercase letters for vehicle treatment, uppercase letters for FK + IBMX treatment).

Previous studies showed that several KLF family members can exert strong influence on neurite outgrowth (Denver et al., 1999; Cayrou et al., 2002; Bonett et al., 2009; Moore et al., 2009; Galvao et al., 2018). KLF9 and KLF13 impact several signaling pathways involved with axon growth and regeneration, including the cAMP signaling pathway (Knoedler et al., 2017; Ávila-Mendoza et al., 2020) whose activation is a hallmark of regenerative responses of neurons to injury (Qiu et al., 2002; Mar et al., 2014). To investigate possible actions of KLF9 and KLF13 on the morphology of hippocampal neurons, and possible roles in modulating cAMP-dependent neurite outgrowth, we analyzed the effects of their forced expression on neurite outgrowth in HT22 cells. Treatment of the control cell line HT22-TR-3 with FK + IBMX for 16 hr increased neurite length by ∼2 fold compared with vehicle treated cells, and co-treatment with dox had no effect on the response to FK + IBMX (Figure 7A). Treatment of the HT22-TR/TO-*Klf9* and HT22-TR/TO-V5*Klf13*-1 cell lines with FK + IBMX (no dox) also increased neurite length, but to a lesser extent than the control cells (1.8 fold and 1.3 fold, respectively), perhaps due to leaky expression of the transgenes. This increase in neurite length caused by FK + IBMX was completely blocked after forced expression of *Klf9* or *V5Klf13* by treatment with dox (Figure 7A). We saw small reductions in baseline neurite length (i.e., without FK + IBMX) caused by dox treatment in HT22-TR-3 (17%) and HT22-TR/TO-*Klf9* (22%) cells, but this effect was larger in the HT22-TR/TO-V5*Klf13*-1 cells (41%). Correcting for the small non-specific effect of dox on neurite length in control cells, forced expression of *Klf9* had no effect on baseline (non-cAMP stimulated) neurite length, while forced expression of *V5Klf13* reduced baseline neurite length by 30%.

We next investigated if KLF9 or KLF13 deficiency can impact cAMP-dependent neurite outgrowth using HT22 cells in which the *Klf9* or *Klf13* genes (or both) were inactivated by CRISPR/Cas9 genome editing (Knoedler et al., 2017, 2020; Ávila-Mendoza et al., 2020). The baseline neurite lengths were greater compared with the parent cell line in *Klf9-*KO (20%), *Klf13*-KO (28%) and *Klf9/13* double-KO (60%) cells (Figure 7B). Treatment with FK + IBMX increased neurite length in all four cell lines, but the absolute neurite lengths were significantly greater compared with the parent cells in the *Klf13*-KO (41%) and *Klf9/13* double-KO (58%) (Figure 7B). The magnitude change in neurite length (the difference between baseline and FK + IBMX) was similar (1.3–1.4 fold) between the four cell lines.

### KLF9 and KLF13 Inhibit Axon Growth in Primary Hippocampal Neurons

**FIGURE 8:**
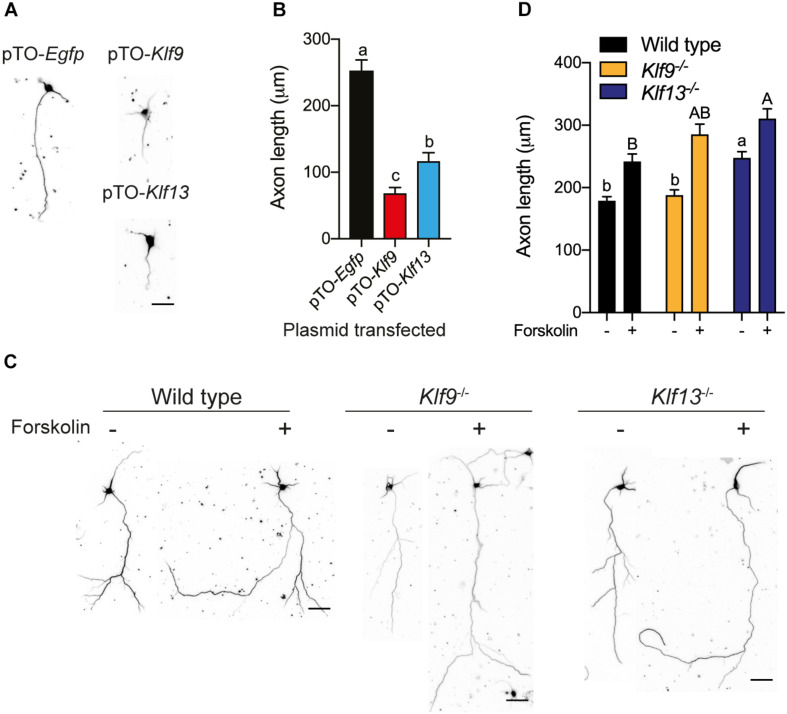
Forced expression of *Klf9* or *Klf13* inhibits axon growth, while inactivation of *Klf9* or *Klf13* enhances cAMP-dependent axon growth in primary hippocampal neurons. We analyzed the effects of forced expression of *Klf9* or *Klf13* on axon growth in primary hippocampal neurons isolated from PND1 wild type mice, and the effects of loss of *Klf9* or *Klf13* on baseline and forskolin (FK)-stimulated axon growth in primary hippocampal neurons isolated from WT, *Klf9*^–/–^ and *Klf13*^–/–^ mice. We conducted immunocytochemistry (ICC) using a monoclonal β-tubulin III antibody and measured β-tubulin-stained projections as described in the section “Materials and Methods.” **(A)** Representative images of primary hippocampal neurons isolated from WT mice and transfected with pTO-*Egfp* (control), pTO-*Klf9* or pTO-*Klf13* vectors. We cultured cells for 3 days after transfection before fixing for ICC. Scale bar = 50 μm. **(B)** Quantification of axon lengths of primary neurons transfected with the indicated vectors. Bars represent the mean ± SEM. Forced expression of *Klf9* or *Klf13* strongly suppressed axon growth [*F*_(2,38)_ = 42.37, *p* < 0.0001; one-way ANOVA followed by Tukey’s *post hoc* test; we analyzed > 100 cells per culture, with *n* = 3/treatment]. Means with the same letter are not significantly different (*p* < 0.05). **(C)** Representative images of primary hippocampal neurons isolated from PND1 WT, *Klf9*^–/–^ and *Klf13*^–/–^ mice treated with vehicle or FK (5 μM) for 4 days before conducting ICC. Scale bars = 50 μm. **(D)** Quantification of axon lengths of primary hippocampal neurons treated with vehicle or FK. Bars represent the mean ± SEM; we analyzed > 100 cells per culture, with *n* = 3/treatment). The baseline axon length was greater in neurons from *Klf13*^–/–^ mice compared with WT mice [*F*_(2,483)_ = 17.29, *p* < 0.0001]. Axon growth was induced by FK treatment in neurons from all genotypes (*p* < 0.05, Student’s independent *t*-test), but the absolute axon length was greater in cells from *Klf13*^–/–^ mice compared with WT or *Klf9*^–/–^ mice [*F*_(2,399)_ = 5.394, *p* = 0.0049]. Means with the same letter are not significantly different (*p* < 0.05) between vehicle (lowercase letters) or FK treatment (uppercase letters).

We also looked at axon growth in primary hippocampal neurons (fixed cells were immunostained for β-tubulin III) isolated from wild type mice, and transfected with the expression plasmids pTO-*Egfp* (control), pTO-*Klf9* or pTO-*Klf13*. Forced expression of *Klf9* reduced axon length by 73% compared with cells transfected with the control vector, while axon length was reduced by 53% after forced expression of *Klf13* (Figures 8A,B).

We next looked at primary hippocampal neurons from wild type and *Klf* deficient mice and found that the baseline axon length was not different in cells from *Klf9*^–/–^ mice compared with wild type, but was greater in cells from *Klf13*^–/–^ mice (Figures 8C,D). Treatment with FK increased axon length in primary neurons isolated from the three genotypes. The absolute axon length after FK treatment was greater in cells from *Klf13*^–/–^ mice compared with wild type (wild type and *Klf9*^–/–^ were not different from each other, nor were *Klf9*^–/–^ and *Klf13*^–/–^). The magnitude change in axon length was similar among the three genotypes (wild type 1.3 fold; *Klf9*^–/–^ 1.5 fold; *Klf13*^–/–^ 1.2 fold).

## Discussion

Here we show that KLF9 and KLF13 inhibit the intrinsic capacity of hippocampal neurons to grow processes, in part by negatively regulating activity of the cAMP signaling pathway. Using the adult mouse hippocampus-derived cell line HT22, we found that KLF9 and KLF13 repress the mRNA levels of several genes involved with cAMP signaling. Notably, KLF13 regulated four times more genes in this pathway than KLF9, and KLF13 was more potent than KLF9 in affecting several other measures of cAMP pathway activity, including neurite extension. We extended our findings in HT22 to primary hippocampal neurons and found that forced expression of *Klf9* or *Klf13* inhibited axon growth, while deficiency of *Klf13* enhanced cAMP-induced axon growth. Taken together, our findings point to pivotal roles for these paralogous KLFs in maintaining the differentiated state of mammalian neurons, and suggest that targeting these TFs could represent an important paradigm for promoting nerve regeneration (Benowitz et al., 2017).

The expression of *Klf9* in the brain begins around embryonic day 12.5, and in the hippocampus and cerebellum this expression increases strongly during the first postnatal month (Morita et al., 2003; Denver and Williamson, 2009). *Klf9* deficiency did not cause gross defects in brain structure under stable physiological conditions, but led to behavioral defects revealed by impaired responses in the rotorod and contextual fear-conditioning tests (Morita et al., 2003). Scobie et al. (2009) showed that hippocampal neurons lacking *Klf9* exhibited delayed maturation, as reflected by altered expression of early phase markers, dendrite formation and electrophysiological properties (Scobie et al., 2009). In the current study we corroborated earlier findings showing a postnatal rise in *Klf9* mRNA in the hippocampus, and we also show a similar profile for *Klf13* mRNA. It is currently unknown whether *Klf13* gene disruption has any effect on development of the central nervous system or on behavior, although *Klf13* knockout mice are viable and show no obvious defects (Heard et al., 2012). The failure of single *Klf9* or *Klf13* knockout to generate strong neural phenotypes may be explained by our recent observations that these two closely related transcription factors can compensate for the loss of the other for certain cellular signaling pathways and functions (Heard et al., 2012; Knoedler et al., 2017, 2020; Ávila-Mendoza et al., 2020). We hypothesize that inactivation of both *Klf* genes will lead to more significant neurodevelopmental effects.

Our previous global analyses of gene expression (RNA-seq) and chromatin occupancy of KLF9 and KLF13 (ChSP-seq) revealed several pathways involved with the dynamics of axon growth and regeneration; the complete KEGG pathway analyses are given in Knoedler et al. (2017) and Ávila-Mendoza et al. (2020). For example, we found “Regulation of actin cytoskeleton” (KEGG:04810), “Axon guidance” (KEGG: 04360), “Neurotrophin signaling pathway” (KEGG: 04722) and “cAMP signaling pathway” (KEGG: 04024) to be affected by both KLF9 and KLF13. There are other pathways impacted by the two KLFs that could be involved indirectly or directly in the regulation of axon growth and regeneration. Notably, many of the pathways affected by KLF9 and KLF13 converge on the cAMP pathway (Knoedler et al., 2017; Ávila-Mendoza et al., 2020). Here we focused on cAMP signaling since it plays a fundamental role in the elaboration of neuronal projections during development (Cai et al., 2001), and it’s activation is a hallmark of regenerative responses of neurons to injury (Qiu et al., 2002; Mar et al., 2014; Batty et al., 2017).

The formation of cAMP is catalyzed by adenylyl cyclases, which leads to a signaling cascade where cAMP acts directly on three main targets: protein kinase A (PKA), the exchange protein activated by cAMP (EPAC), and cyclic nucleotide-gated ion channels (CNGCs). These proteins in turn modulate several cellular substrates that function as pathway effectors, including the cAMP response element-binding proteins (Fimia and Sassone-Corsi, 2001). Analysis of our previous RNA-seq datasets showed that KLF9 and KLF13 can repress several genes in the cAMP signaling cascade, and targeted analyses in the current study confirmed this regulation for the *Prkaca*, *Rapgef3* (a.k.a. *Epac1*), *Rap1a* (which is a substrate of EPAC), *Calm3*, *Adcy6*, *Tiam1*, and *Vav2* genes.

Using targeted ChIP-qPCR assays in HT22-TR/TO-*Klf9* and HT22-TR/TO-*V5Klf13*-1 cells treated with dox we confirmed association in chromatin for KLF9 at the *Prkaca* and *Rapgef3* proximal promoter, and KLF13 at the *Prkaca*, *Rap1a*, *Calm3, Adcy6*, and *Rapgef3* proximal promoters. The *Klf16* promoter, which we previously found to have large and broad ChSP-seq peaks and robust ChIP signals, and was strongly repressed by both KLFs, served as a positive control. Using transfection-reporter assays we found that the repression of two of the cAMP pathway genes, *Prkaca* and *Calm3*, is likely through direct transcriptional repression; transcriptional activity of the *Prkaca* promoter was repressed by forced expression of either *Klf9* or *V5Klf13*, while the *Calm3* promoter activity was repressed by expression of *V5Klf13*. Furthermore, the gene repression caused by the KLFs correlated with suppression of cAMP signaling in cells, evidenced by our transfection-reporter assays using a cAMP-responsive reporter construct.

During development of the mouse central nervous system the concentration of cAMP is highest in embryonic neurons, then declines rapidly within the first 5 days of postnatal life, and remains low in adulthood (Cai et al., 2001). This suggests that there is greater activity of the pathway during embryonic development coinciding with its roles in neurogenesis, cell survival, differentiation, neurite outgrowth and synaptogenesis (Averaimo and Nicol, 2014; Nicol and Gaspar, 2014; Kelly, 2018). Conversely, we found that the levels of *Klf9* and *Klf13* mRNAs increased during postnatal development and remained elevated in adulthood, which agrees with previous reports (Denver et al., 1999; Denver and Williamson, 2009; Moore et al., 2009). The inverse relationship between developmental changes in cAMP and the expression of the *Klf* genes provides additional support for the hypothesis that KLF9 and KLF13 play a role in the postnatal repression of the cAMP signaling pathway in neurons.

We confirmed that KLF13 associates in chromatin at the *Prkaca* promoter region in hippocampus of PND30 mice, but we did not see enrichment for either KLF at the *Rap1a*, *Calm3* or *Adcy6* genes. This may indicate that the HT22 cells do not fully recapitulate the situation in hippocampal neurons *in vivo*. On the other hand, the failure to find KLF association in chromatin at these genes may reflect limitations of the experimental design and/or the assays. For example, we analyzed only a single timepoint (animals were sacrificed between 9 and 11 a.m.) and we know that *Klf* genes undergo circadian oscillation in their transcription and KLF association in chromatin throughout the day (Spörl et al., 2012; Yoshitane et al., 2014; Knoedler et al., 2020); a thorough time course experiment will be necessary to address this issue. Also, the KLFs may differ in their regulation of cAMP pathway genes *in vivo* depending on the physiological or behavioral state. Lastly, the findings may have been affected by the sensitivity of our ChIP assays, which relied on analyzing a section of brain tissue comprised of many cell types with different chromatin states and transcriptional activity, unlike the situation in the HT22 cells.

We found that forced expression of *Klf9* or *Klf13* blocked cAMP-dependent neurite growth in HT22 cells, and axon elaboration in primary hippocampal neurons. Whereas, *Klf9* or *Klf13* deficiency enhanced the effects of cAMP on neuronal morphology, both in HT22 and primary cells. The inhibitory effects of some KLFs on axon growth were initially studied by Moore et al. (2009) who showed that forced expression of several KLFs in cortical neurons (including KLF9 and KLF13) inhibited axon growth. Our results expand on these findings and provide evidence that the inhibitory actions of these two KLFs may be mediated in part by inhibition of the cAMP signaling pathway. Protein kinase A is a central component of this pathway, and we found that transcription of the PKA catalytic subunit A was repressed by both KLFs. In addition, the exchange proteins directly activated by cAMP (EPACs) work cooperatively with PKA and play fundamental roles in neurite elongation mediated by cAMP (Murray and Shewan, 2008; Murray et al., 2009; Emery et al., 2017). We discovered that the *Rapgef3* gene that encodes an EPAC1 variant was also repressed by both KLFs, although we only found a ChSP-seq peak here for KLF9. This may indicate that the actions of KLF13 on this gene are indirect, or mediated through a KLF13 binding site in DNA that is distant from the *Rapgef3* promoter; e.g., perhaps one of the KLF13 peaks in adjacent genes communicates with the *Rapgef3* promoter through chromosome looping. The gene encoding calmodulin 3 was directly repressed by KLF13, supporting that multiple arms of the pathway are affected by the KLFs. It is well known that elevated levels of cAMP (Cai et al., 2001; Qiu et al., 2002; Batty et al., 2017; Inda et al., 2017) and the consequent activation of both PKA and EPAC (Murray and Shewan, 2008) are associated with improved neurite outgrowth capacity of neurons of the central nervous system. Thus, repression of the cAMP pathway by the KLFs likely plays a role in their suppression of neurite outgrowth.

Additional mechanisms are likely also involved in the inhibitory actions of KLF9 and KLF13 on neurite growth. For example, our previous RNA-seq and ChSP-seq analyses revealed that the gene encoding the dual-specificity phosphatase 14 (DUSP14) enzyme is directly induced by KLF13 (Ávila-Mendoza et al., 2020). DUSP14 is regulated by KLF9 in retinal ganglion cells and functions as a downstream effector of KLF9 in the suppression of axon growth (Galvao et al., 2018). A similar mechanism might operate for KLF13 (and KLF9) in hippocampal neurons. KLF16, which also has inhibitory actions on neurite outgrowth (Moore et al., 2009) and is also member of KLF subfamily 3, transactivates the gene encoding the ephrin receptor *EphA5*, which when activated by its ligand promotes growth cone collapse and consequently the inhibition of axon growth of retinal ganglion cells (Wang et al., 2016). Our previous work showed that KLF13, like KLF16, induces the *EphA5* gene (Wang et al., 2016; Ávila-Mendoza et al., 2020) and may therefore also support inhibition of axon growth via this cell surface signaling mechanism. It is also noteworthy that KLF9 and KLF13 repress genes that encode cytoskeletal proteins, which likely contributes to their inhibition of neuronal process formation (Knoedler et al., 2017; Ávila-Mendoza et al., 2020).

Our results showing that single knockout of *Klf9* or *Klf13* leads to increased baseline neurite length, and enhanced cAMP-dependent neurite growth in HT22 cells suggest that these KLFs cooperate to regulate cellular pathways controlling neuronal morphology and circuit formation. Silencing of *Klf9* in retinal ganglion cells improved axon growth *in vitro*, and optic nerve regeneration after injury *in vivo* (Apara et al., 2017; Galvao et al., 2018). To our knowledge, similar studies have not yet been conducted for *Klf13*. The effect that manipulating the two *Klfs* had on the cell morphological responses to elevated cAMP may be explained by their repression of key components of this pathway like EPAC and PKA, among others. We saw larger and more consistent effects on cAMP-induced neurite outgrowth in HT22, and axon extension in primary neurons when we manipulated *Klf13* compared with *Klf9*. This may be explained by our observation that KLF13 regulates four times more cAMP pathway genes than KLF9. Nevertheless, our finding that these effects were greater in double KO compared with single KO cells supports that the two KLFs play cooperative roles in regulating neurite/axon growth. There is mounting evidence for cooperative and compensatory actions of KLFs in different cell types for different cellular functions, supporting that evolution has produced redundancy in the pathways regulated by KLFs to provide robustness to their regulation of intracellular signaling pathways (Jiang et al., 2008; Wang et al., 2016; Galvao et al., 2018; Knoedler et al., 2020).

While we focused here on the paralogs KLF9 and KLF13, it is important to recognize that several other KLFs (KLFs 1, 2, 4, 5, 14, 15, 16) have repressive effects on neurite outgrowth, while others (KLFs 6, 7) induce neurite outgrowth (Moore et al., 2009). Also, the KLFs form a transcriptional regulatory network that modulates KLF activities in cells, with both cross- and autoregulation occurring (Dang et al., 2002; Eaton et al., 2008; Pabona et al., 2010; Heard et al., 2012; Knoedler and Denver, 2014; Knoedler et al., 2017; Ávila-Mendoza et al., 2020). Cross-regulation among the KLFs likely modulates their expression levels appropriate to the specific developmental stage or physiological state (Knoedler and Denver, 2014).

**FIGURE 9:**
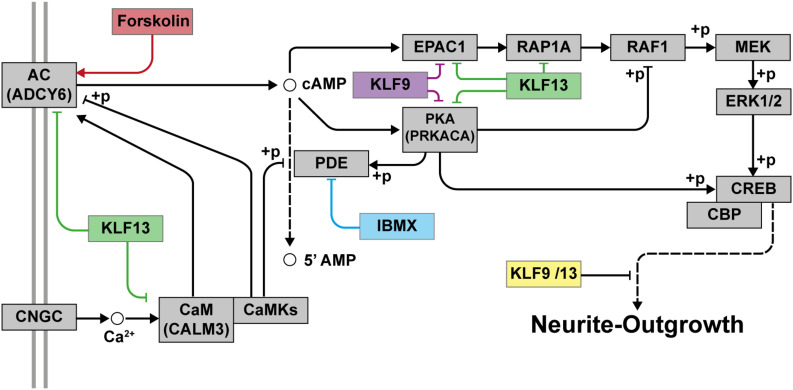
Proposed model for the repressive actions of KLF9 and KLF13 on cAMP-dependent neurite-outgrowth. Neurite-outgrowth is promoted by activation of the cAMP signaling pathway. Experimentally, we used forskolin to activate adenylate cyclase (AC) and 3-isobutyl-1-methylxanthine (IBMX) to inhibit phosphodiesterase. KLF9 and/or KLF13 repress the expression of several genes of the cAMP signaling pathway. Shown in the figure are the proteins that would be affected by KLF9 and KLF13 gene repression. Adenylate cyclase 6 (ADCY6), calmodulin 3 (CALM3), protein kinase A alpha subunit (PRKACA) and Rap guanine nucleotide exchange factor 3 (also known as EPAC1). CNGC: cyclic nucleotide gated channel; CaM: calmodulin; CaMKs: calmodulin-dependent kinases; PKA: protein kinase A; RAP1A: Ras-related protein 1a; RAF1: v-raf-leukemia viral oncogene 1; MEK: mitogen-activated protein kinase kinase; ERK1/2: extracellular signal-regulated kinases 1 and 2; CREB: cAMP response element-binding protein; CBP: CREB-binding protein; +p: phosphorylation.

Taken together, our findings support that KLF9 and KLF13 deficiency generates a permissive state in adult mammalian neurons that allows for significant process elaboration when cell surface signaling pathways are activated (Figure 9). Targeting these and other KLFs, in addition to treatment with regeneration-stimulating factors, may represent a means to promote axon regeneration in humans after nerve injury; e.g., optic nerve regeneration in retina ganglion cells damaged by different retinopathies such as glaucoma or inducing the regeneration of axons after spinal cord injuries.

## Data Availability Statement

The raw data supporting the conclusions of this article will be made available by the authors, without undue reservation.

## Ethics Statement

The animal study was reviewed and approved by Institutional Animal Care and Use Committee at the University of Michigan.

## Author Contributions

JA-M conceived of the project, designed and conducted the experiments, analyzed the data, and wrote the manuscript. AS conducted the experiments, analyzed the data, and edited the manuscript. RD conceived of the project, secured the funding, designed the experiments, and edited the manuscript.

## Conflict of Interest

The authors declare that the research was conducted in the absence of any commercial or financial relationships that could be construed as a potential conflict of interest.

## Footnotes

1 https://www.ncbi.nlm.nih.gov/tools/primer-blast/
