# Supplementary figures and images for: Krüppel-Like Factors 9 and 13 Block Axon Growth by Transcriptional Repression of Key Components of the cAMP Signaling Pathway
Source: Front Mol Neurosci. 2020 Nov 12;13:602638. doi: 10.3389/fnmol.2020.602638 (PMC7689098; doi:10.3389/fnmol.2020.602638)

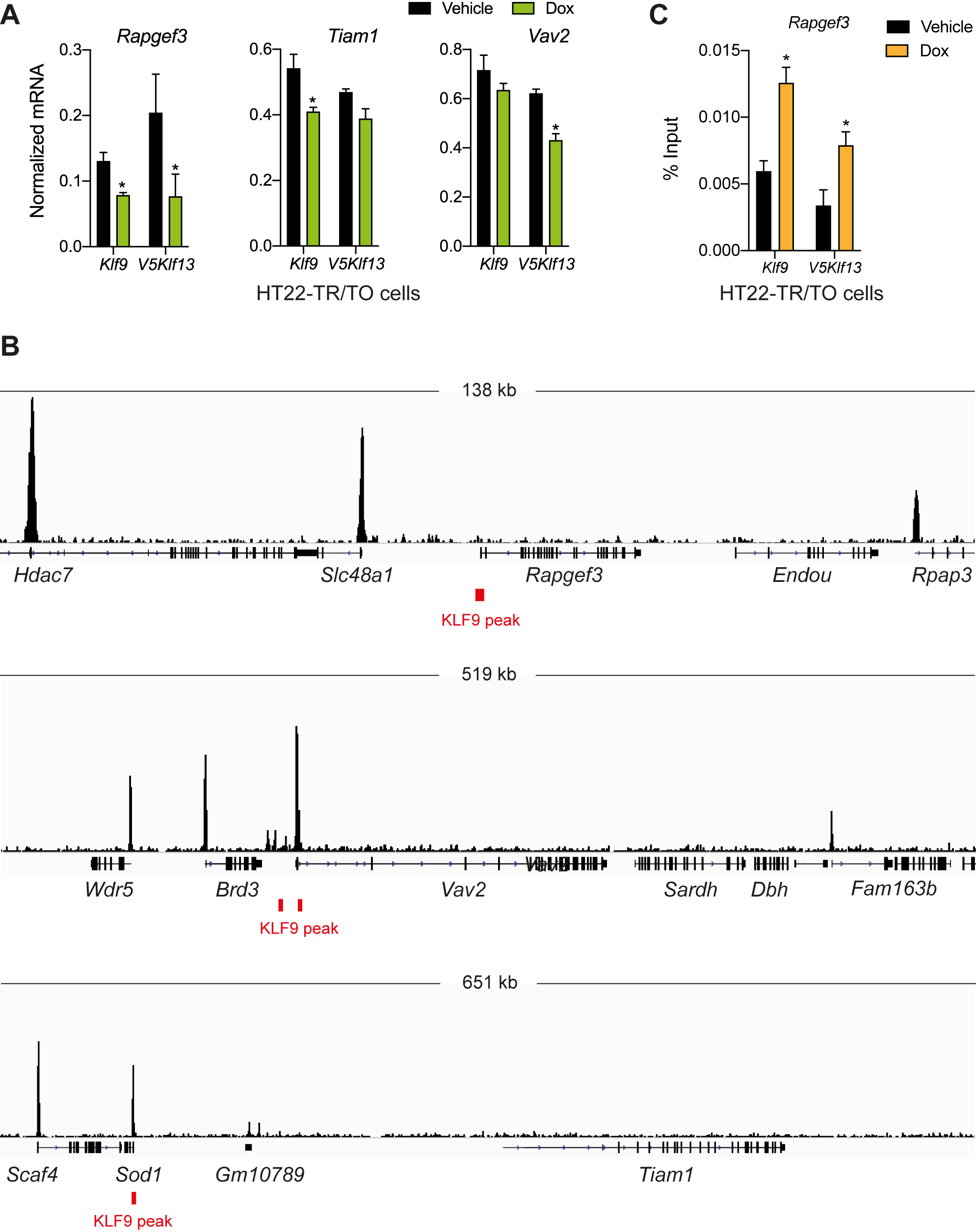

Supplement: Supplementary file 1 [file Image_1.TIF]
